# Emerin preserves stem cell survival through maintenance of centrosome and nuclear lamina structure

**DOI:** 10.1242/dev.204219

**Published:** 2024-11-13

**Authors:** Samuel D. Jones, Jack E. B. Miller, Madilynn M. Amos, Julianna M. Hernández, Katherine M. Piaszynski, Pamela K. Geyer

**Affiliations:** Department of Biochemistry and Molecular Biology, University of Iowa, Iowa City, IA 52242, USA

**Keywords:** Nuclear structure, Lamin, Otefin, Centrosome, Asymmetric division, LEM domain, Polo

## Abstract

*Drosophila* female germline stem cells (GSCs) complete asymmetric mitosis in the presence of an intact, but permeable, nuclear envelope and nuclear lamina (NL). This asymmetric division requires a modified centrosome cycle, wherein mitotic centrosomes with mature pericentriolar material (PCM) embed in the NL and interphase centrosomes with reduced PCM leave the NL. This centrosome cycle requires Emerin, an NL protein required for GSC survival and germ cell differentiation. In *emerin* mutants, interphase GSC centrosomes retain excess PCM, remain embedded in the NL and nucleate microtubule asters at positions of NL distortion. Here, we investigate the contributions of abnormal interphase centrosomes to GSC loss. Remarkably, reducing interphase PCM in *emerin* mutants rescues GSC survival and partially restores germ cell differentiation. Direct tests of the effects of abnormal centrosomes were achieved by expression of constitutively active Polo kinase to drive enlargement of interphase centrosomes in wild-type GSCs. Notably, these conditions failed to alter NL structure or decrease GSC survival. However, coupling enlarged interphase centrosomes with nuclear distortion promoted GSC loss. These studies establish that Emerin maintains centrosome structure to preserve stem cell survival.

## Teaser

**Summary:** The nuclear lamina protein Emerin plays a dual role in the survival of *Drosophila* germline stem cells by maintaining nuclear lamina structure and ensuring reduction of pericentriolar material.

## INTRODUCTION

Stem cells serve as a reservoir of differentiating cells that replace damaged cells to maintain tissue homeostasis (Schultz and Sinclair, 2016). Preservation of these reserves results from asymmetric mitotic divisions of stem cells that generate daughters of different cell fates, one that self-renews and one that differentiates. Such distinct cell fates depend upon the polarized distribution of cell fate determinants, achieved by precise alignment of the plane of mitotic division (Venkei and Yamashita, 2018). As a consequence, defects in components of the mitotic apparatus can impact the balance of self-renewing and differentiating daughters, leading to declines in stem cell populations and tissue health.

Germline stem cells (GSCs) in the *Drosophila* ovary represent an excellent model for investigating the mechanisms associated with asymmetric cell division and stem cell homeostasis (Kahney et al., 2019; Venkei and Yamashita, 2018). Ovaries are divided into 16-20 ovarioles that each contain an anterior germarium comprising somatic niche cells that anchor two to three GSCs (Hinnant et al., 2020; Spradling et al., 2011). The somatic niche establishes a signaling environment required for GSC homeostasis. Central to this regulation are BMP ligands that are secreted by somatic niche cells and received by GSCs to promote a GSC transcriptional program that includes repression of the key differentiation gene *bag of marbles* (Xie and Li, 2007). Although niche signals have a dominant role in GSC maintenance (Nelson et al., 2019), the uneven distribution of intrinsic nuclear factors serves an additional layer of regulation that ensures a binary outcome from a single mitotic division (Fichelson et al., 2009; Zhang et al., 2014). Crucially, optimized exposure of GSCs and daughters to extrinsic and intrinsic regulatory factors depends upon the plane of the asymmetric mitotic division, defined by the orientation of the mitotic spindle. In female GSCs, the mitotic spindle is positioned perpendicular to the niche and anchored to the spectrosome, a germline-specific organelle that localizes to the interface between GSCs and niche cells (Lin et al., 1994). Spindle orientation is achieved through spectrosome recruitment of centrosomes (Lu et al., 2012; Stevens et al., 2007; Villa-Fombuena et al., 2021), so that the mitotic division will generate one daughter GSC that remains at the niche interface and self-renews and one daughter cystoblast (CB) that is displaced from the niche and differentiates. CB differentiation includes four synchronous, incomplete mitotic divisions to form an interconnected 16-cell cyst. Within this cyst, one germ cell enters meiosis and becomes the oocyte, and the remaining 15 germ cells become nurse cells that support the oocyte. These early stages of germ cell development emphasize the importance of an existing intracellular polarity in GSCs that directs the position of the mitotic spindle for execution of an asymmetric division that promotes GSC homeostasis.

Asymmetric mitosis in female GSCs is specialized. In contrast to somatic cells, which undergo nuclear envelope breakdown, GSC mitosis occurs in the presence of a permeable, but intact, nuclear envelope and underlying nuclear lamina (NL) network (Duan et al., 2021). In this ‘semi-closed’ mode, mitotic spindles are nucleated within the confines of the nucleus, and centrosomes gain access to the nuclear interior through their insertion into a cup-like structure within a remodeled NL, a structure that resembles the *Caenorhabditis elegans* centriculum (Maheshwari et al., 2023). ‘Semi-closed’ mitosis requires that centrosomes engage in a modified maturation cycle. In GSCs, as in many *Drosophila* cell types, interphase centrosomes comprise a pair of centrioles surrounded by a thin pericentriolar material (PCM) layer that is unable to nucleate microtubules (Rogers et al., 2008; Stevens et al., 2007). As cells progress into mitosis, centrosomes mature, whereby centrioles dramatically increase PCM levels to produce active centrosomes that nucleate substantial amounts of microtubule asters (Palazzo et al., 2000). Upon mitotic exit, PCM levels are reduced and centrosomes become largely inactive. The specialized ‘semi-closed’ mitosis in GSCs requires that the centrosome maturation cycle is coupled with centrosome movement into and out of the NL. This movement requires NL proteins, including the NL LEM-domain protein Emerin (also known as Otefin; Duan et al., 2021). In *emerin* mutants, reduction of PCM at the end of mitosis is impaired, resulting in centrosomes that carry excess PCM and nucleate aster microtubules (Duan et al., 2021). These centrosome defects are coupled with decreased GSC survival and blocked germ cell differentiation (Duan et al., 2021). Such findings suggest that the specialized mode of asymmetric mitosis in GSCs sensitizes these stem cells to defects in NL proteins.

GSC death in *emerin* mutants results from activation of a GSC checkpoint that depends upon two DNA damage response (DDR) kinases, Ataxia-telangiectasia and RAD3-related protein (ATR; also known as Mei-41) and Checkpoint kinase 2 (Chk2; also known as Lok) (Barton et al., 2018). Mechanisms responsible for checkpoint activation in *emerin* mutants are poorly understood. Whereas Emerin loss reduces mitotic integrity and increases DNA damage in GSCs (Duan et al., 2021), neither defect appears to trigger the checkpoint pathway. Indeed, depletion of the NL scaffolding protein Lamin B (LamB; also known as Lam and Lamin Dm0) causes greater chromosome segregation defects than Emerin loss, yet GSCs survive and germ cells differentiate (Duan et al., 2021). Additionally, DNA damage is absent in *chk2, emerin* double-mutant GSCs, establishing that DNA damage occurs downstream of Chk2 activation (Barton et al., 2018). Here, we investigated the connection between abnormal centrosome structure and checkpoint activation. Active centrosomes assemble large protein machines that contribute to diverse cellular processes such as intracellular transport, organelle distribution, and cell shape (Arquint et al., 2014; Lattao et al., 2017; Rusan and Rogers, 2009). As such, an altered microtubule network in *emerin* mutants might be responsible for decreased health and survival of GSCs. We tested whether centrosomes that carry excess PCM are necessary and sufficient to disrupt germ cell function. Remarkably, we found that decreased levels of interphase PCM in *emerin* mutants rescues GSC viability and partially restores germ cell differentiation, revealing that centrosome defects contribute to GSC loss. Although decreased PCM reduced microtubule enrichment at *emerin* mutant interphase centrosomes, the NL remained distorted, demonstrating that nuclear deformation is not caused by nucleation of microtubules within the NL. Next, we generated abnormal interphase centrosomes in wild-type GSCs, by expressing a constitutively active Polo kinase variant that drove PCM enlargement. Notably, these conditions did not alter nuclear structure or decrease GSC survival, suggesting that the presence of abnormal centrosomes alone does not promote GSC loss. Finally, we simultaneously induced enlarged interphase centrosomes and severe NL distortion in wild-type GSCs. We found that this compromised GSC survival and germ cell differentiation, reproducing *emerin* mutant phenotypes. Based on these observations, we conclude that activation of the GSC checkpoint requires two inputs, one corresponding to defects in interphase centrosome structure and one corresponding to NL distortion. Collectively, our studies identify failures in the centrosome cycle as a new contributor to stem cell loss resulting from mutation of an NL component.

## RESULTS

### PCM reduction rescues *emerin* mutant GSC loss

To test the role of abnormal interphase centrosomes in GSC loss, we genetically reduced PCM levels in *emerin* mutants and assessed effects on GSC survival. To this end, we generated *centrosomin* (*cnn*)*, emerin* and *emerin; spindle defective-2* (*spd-2*) double mutants. We chose null mutations in genes encoding Cnn and Spd-2 because these proteins are major PCM scaffolding proteins (Conduit et al., 2014). Indeed, mitotic PCM builds by Spd-2 recruitment of Cnn to the centriole, where Polo kinase phosphorylates Cnn to promote a feed-forward loop that increases PCM assembly. Mitotic PCM expansion docks the γ-tubulin ring complexes to nucleate microtubules (Conduit et al., 2015). We reasoned that if excess PCM and active centrosomes contribute to GSC loss and blocked differentiation, then reduced levels of PCM scaffolding proteins would reverse these mutant phenotypes.

Females carrying null alleles of *cnn* and *spd-2* are sterile due to the essential requirements for these PCM proteins in embryogenesis (Giansanti et al., 2008; Megraw et al., 1999; Vaizel-Ohayon and Schejter, 1999). However, we predicted that loss of Cnn or Spd-2 would be inconsequential to GSC survival, as centrosomes are dispensable for oogenesis (Stevens et al., 2007). To test this prediction, we examined effects of loss of either Cnn or Spd-2 on oogenesis. To this end, ovaries were dissected and stained with antibodies against Engrailed, a transcription factor expressed at high levels in somatic cells in the niche and in differentiating egg chambers (Forbes et al., 1996), and Vasa, a germ cell-specific helicase expressed in all germ cells (Lasko and Ashburner, 1988). Immunohistochemical analysis of newly eclosed and 14-day-old *cnn* or *spd-2* mutant ovaries demonstrated that all germaria in ovaries of both ages and genotypes had germ cells and all germaria were attached to strings of differentiating egg chambers (Fig. S1A). We also stained newly eclosed and 14-day-old *cnn* or *spd-2* mutant ovaries with antibodies against Vasa and Spectrin, a cytoskeletal protein that shows a distinct subcellular localization depending on cell type. In somatic cells, Spectrin localizes to the cellular periphery. In germ cells, Spectrin forms a spherical structure called a spectrosome in GSCs and branches to form a fusome in differentiating germ cells (Lin et al., 1994). Immunohistochemical analysis of newly eclosed and 14-day-old *cnn* or *spd-2* mutant ovaries showed that all germaria in ovaries dissected from females of both ages and genotypes had germaria with branched fusomes (Fig. S1B), indicating that germ cell differentiation occurs in the absence of these PCM proteins. We conclude that Cnn and Spd-2 are dispensable for oogenesis.

**Fig. 1.**
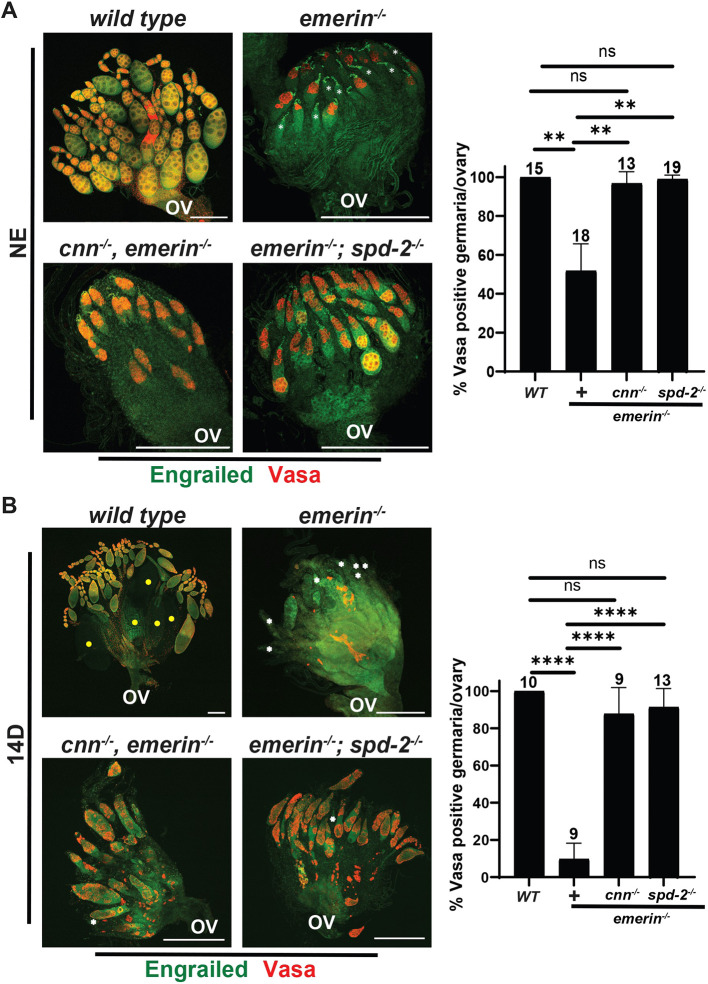
**Loss of Cnn or Spd-2 rescues stem cell death in *emerin* mutants.** (A,B) Left: Confocal images of newly eclosed (NE; A) or 14-day (14D; B) ovaries of the indicated genotypes stained with antibodies against Engrailed (green) and Vasa (red). Ovaries are oriented with anterior (germaria) at the top and posterior (oviduct, OV) at the bottom. White asterisks identify Vasa-negative germaria that do not contain germ cells. Yellow circles show late-stage egg chambers. Scale bars: 200 µm. Right: Bar graph quantification of the percentage of germaria per ovary that contain germ cells. Genotypes are shown along the *x*-axis, with ‘+’ indicating the *emerin^−/−^* line. The number of ovaries analyzed is indicated at the top of the bars. Statistical analysis used the unpaired, two-sample *t*-test. **P<0.01; ****P<0.0001. ns, not significant.

We next evaluated phenotypes of *cnn, emerin* and *emerin; spd-2* mutant ovaries. Newly eclosed *emerin* single- and double-mutant ovaries were stained with antibodies against Engrailed and Vasa. In newly eclosed *emerin* mutant ovaries, germ cell loss was apparent, evidenced by a reduction in the number of germaria that carried Vasa-positive cells (51% versus 100% in wild-type ovaries; Fig. 1A). In contrast, nearly all *cnn, emerin* and *emerin; spd-2* mutant germaria had germ cells (97% and 99% of ovarioles, respectively). Even so, long strings of differentiating egg chambers were largely absent from double-mutant ovaries, with only a few germaria attached to one or two poorly differentiating egg chambers (Fig. 1A). These data reveal a striking suppression of germ cell loss without a strong rescue of differentiation. To understand the extent of rescued survival, we stained 14-day-old ovaries with Engrailed and Vasa antibodies (Fig. 1B). Few (10%) 2-week-old *emerin* mutant germaria carried GSCs. In contrast, 14-day-old old *cnn, emerin* or *emerin; spd-2* mutant ovaries had germ cells in nearly all (88% and 91%, respectively) germaria (Fig. 1B), demonstrating sustained survival. We conclude that excess PCM contributes to GSC dysfunction in *emerin* mutants.

### Reduced PCM promotes early differentiation in *emerin* mutant germ cells

**Fig. 2.**
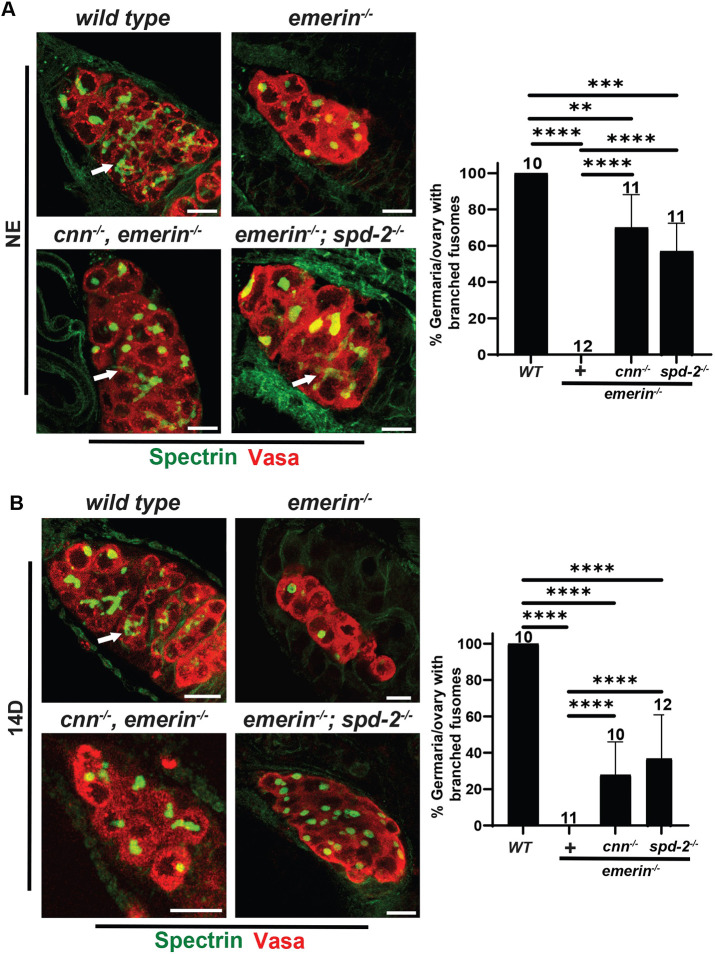
**Germ cells enter differentiation upon PCM reduction in *emerin* mutants.** (A,B) Left: Confocal images of NE (A) and 14-day (14D; B) germaria stained with antibodies against Spectrin (green) and Vasa (red). Germaria are oriented so that anterior is at the top and posterior is at the bottom. White arrows show locations with branched Spectrin. Scale bars: 10 µm. Right: Bar graphs quantifying the percentage of germaria per ovary that carry germ cells with branched fusomes, evidenced by non-spherical Spectrin staining. The number of ovaries analyzed is indicated at the top of the bars. Statistical analysis used the unpaired, two-sample *t*-test. *P<0.01; **P<0.001; ****P<0.0001.

The phenotype of *cnn, emerin* or *emerin; spd-2* double-mutant ovaries suggested that improved germ cell survival was not accompanied with normal differentiation (Fig. 1). Nonetheless, double-mutant germaria appeared larger than *emerin* mutants, indicating that germ cells might have entered early stages of differentiation. For this reason, we stained newly eclosed *emerin* single- and double*-*mutant ovaries with Vasa and Spectrin. In *emerin* single mutants, Spectrin was found only in spectrosomes and never branches (Fig. 2A). In contrast, in *emerin* double-mutant ovaries, anterior germ cells carried spectrosomes and posterior germ cells commonly had branched Spectrin-positive structures (Fig. 2A). Quantification showed that 70% of *cnn, emerin* and 57% of *emerin; spd-2* double-mutant germaria had branched fusomes (Fig. 2A). These data suggest that many double-mutant germ cells initiate differentiation. Nonetheless, 16-cell cysts were rarely formed, implying that differentiation did not progress beyond early stages. We also examined differentiation in 14-day-old *emerin* single- and double-mutant ovaries. Unexpectedly, in aged ovaries of the double mutants, the number of germaria carrying branched fusomes was lower in *cnn, emerin* or *emerin; spd-2* mutants (28% and 37%, respectively; Fig. 2B). We infer that differentiating double-mutant germ cells are unhealthy and are lost. Taken together, we conclude that upon reduction of PCM, *emerin* mutant germ cells enter early stages of germ cell differentiation but fail to progress.

### NL distortion remains in *emerin* mutants lacking activated interphase centrosomes

**Fig. 3.**
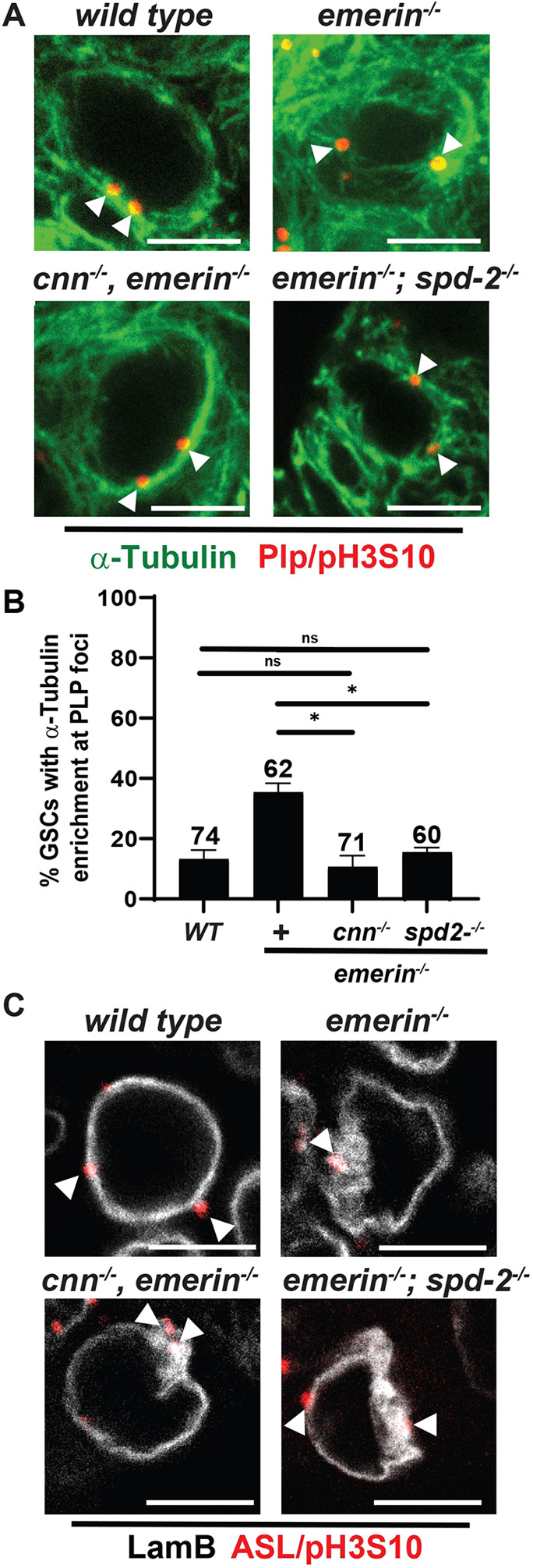
**PCM reduction reverses microtubule enrichment at centrosomes, but not NL distortion in *emerin* mutant GSCs.** (A) Confocal images of <1-day-old interphase germline stem cells (GSCs) of the indicated genotype stained with antibodies against α-Tubulin (green), Pericentrin-like protein (Plp; red), and Histone H3 phosphorylated on S10 (pH3S10; red). White arrowheads indicate centrioles. Scale bars: 5 μm. (B) Bar graph quantification of the percentage of GSCs with centrioles (marked by Plp) showing microtubule enrichment. The total number of GSCs analyzed is listed above each bar. WT, wild type*.* Statistical analysis used the unpaired, two-sample *t*-test. *P<0.05. ns, not significant. (C) Confocal images of <1-day-old GSC nuclei of the indicated genotypes stained with antibodies against LamB (white), Asterless (ASL; red), and pH3S10 (red). White arrowheads point to centrioles. Scale bars: 5 µm. The absence of pH3S10 staining in A and C identified interphase GSCs.

A partial suppression of germ cell phenotypes in the *cnn, emerin* and *emerin; spd-2* double mutants raised the possibility that residual microtubule-nucleating activity remained upon depletion of one of the PCM scaffolding proteins. To test this possibility, we stained double-mutant ovaries with antibodies against Pericentrin-like protein (Plp) (a centriole bridge protein), α-Tubulin (a microtubule component), and phospho-Histone H3 (pH3S10) (a marker of condensed mitotic chromosomes used to identify non-dividing GSCs). In wild-type GSCs, the amount of PCM present around most centrioles was insufficient for microtubule nucleation, evident by tubulin enrichment at only (13%) of interphase centrioles marked by Plp localization (Fig. 3A). This low level of tubulin enrichment likely corresponds to late G2 phase GSCs (still pH3S10 negative) that have initiated centrosome enlargement and are nearly ready to enter mitosis. In *emerin* mutants, interphase centrosomes are commonly active in GSCs, with 35% showing tubulin enrichment and forming microtubule asters at mutant centrioles (Fig. 3A,B). Strikingly, in *cnn, emerin* and *emerin; spd-2* mutant interphase GSCs, the microtubule network was dispersed (Fig. 3A). Quantification demonstrated that tubulin enrichment at centrioles was close to wild-type levels, reduced to 11% (*cnn, emerin*) or 15% (*emerin; spd-2*) in the absence of the PCM scaffolding protein (Fig. 3B). These analyses confirm that loss of Cnn or Spd-2 prevents the majority of *emerin* mutant interphase centrosomes from functioning as microtubule-organizing centers.

Active interphase centrosomes localize to positions of severe NL distortion in *emerin* mutants (Duan et al., 2021). Based on these observations, we predicted that nuclear deformation was linked to enrichment of microtubules at the nuclear periphery. The absence of tubulin enrichment at centrosomes in *cnn, emerin* or *emerin; spd-2* mutants provided an opportunity to test this prediction, with the expectation that structural deformation of the NL would be resolved in the *emerin* double mutants. We stained *cnn, emerin* or *emerin; spd-2* mutant ovaries with antibodies against LamB, the only lamin subtype expressed in GSCs (Perales et al., 2024b), Asterless (Asl) (a centriole bridge protein) (Varadarajan and Rusan, 2018), and pH3S10. Surprisingly, NL distortion remained (Fig. 3C). These data indicate that activated interphase centrosomes are not responsible for the prominent structural changes that occur in *emerin* mutant GSC nuclei.

### Reduction of PCM fails to suppress Chk2 activation in *emerin* mutants

Germ cell loss in *emerin* mutants results from activation of a GSC checkpoint that uses the DDR kinases ATR and Chk2 (Barton et al., 2018; Duan et al., 2020). We wondered whether DDR signaling remained active in the double mutants. To test this possibility, we generated *chk2^+/−^, emerin^−/−^; spd-2^−/−^* mutant females, based on our previous demonstration that loss of one wild-type *chk2* allele rescued fecundity of *emerin* mutant females (Barton et al., 2018). Newly eclosed ovaries were stained with DAPI and antibodies against Vasa. We found that *chk2^+/−^, emerin^−/−^; spd-2^−/−^* mutant ovaries carried strings of differentiating egg chambers (Fig. S2). To evaluate the level of differentiation, we quantified the number of germaria that were attached to at least two contiguous egg chambers. This quantification showed that the majority (64%) of ovarioles were differentiating (Fig. S2). Although *chk2^+/−^, emerin^−/−^; spd-2^−/−^* mutant females laid eggs, the eggs failed to hatch, consistent with previous demonstrations that Emerin and Spd-2 are required for embryogenesis (Barton et al., 2018; Giansanti et al., 2008). Together, these data imply that Chk2 remains active in *cnn, emerin* and *emerin; spd-2* mutant germ cells.

### Ectopic interphase centrosomes do not perturb oogenesis

**Fig. 4.**
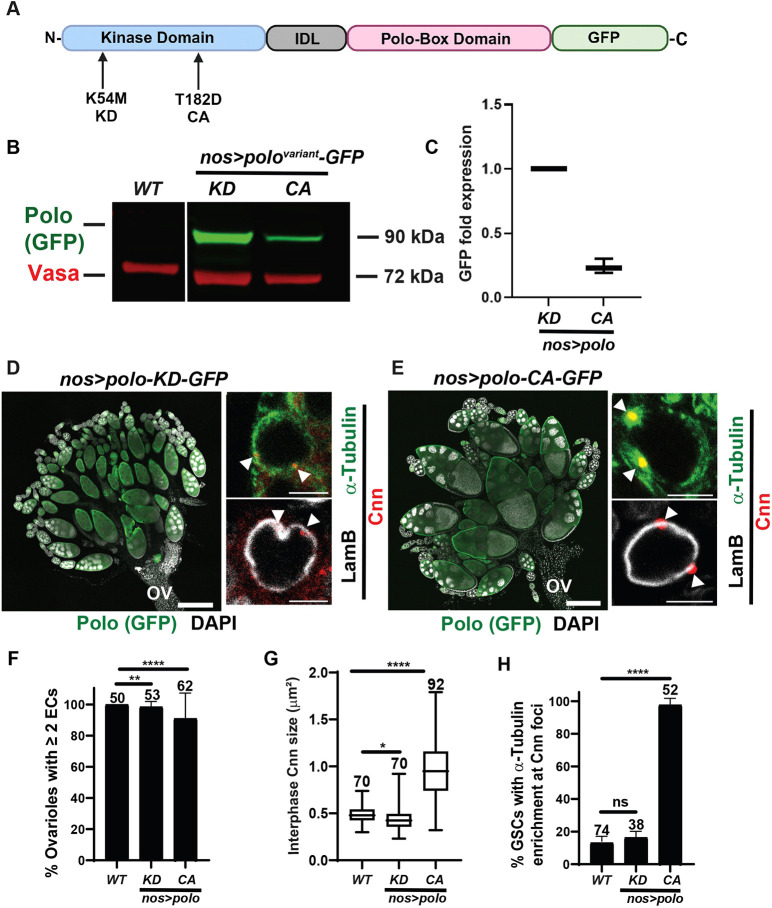
**Oogenesis is robust in the presence of ectopically enlarged interphase centrosomes.** (A) Schematic of the protein structure of the two Polo variants used in these studies. Both variants carry missense mutations in the kinase domain, corresponding to either to K54M (kinase dead, KD) or the phosphomimetic T182D (constitutively active, CA). IDL, inter-domain linker. (B) Western blot of proteins extracted from NE ovaries, probed with antibodies against GFP (Polo, green) and Vasa (red). (C) Western blot quantification of expressed protein levels of Polo variants. Levels were normalized to the Polo-KD-GFP variant. (D,E) Confocal images of <1-day-old *nos>polo-KD-GFP* (D) or *nos>polo-CA-GFP* (E) ovaries and GSC nuclei. Ovaries were stained with DAPI (white) and antibodies against GFP (Polo, green). Ovaries are oriented with anterior (germaria) at the top left and posterior (oviduct, ov) at the bottom right. Scale bars: 200 μm. GSCs were stained with antibodies against α-Tubulin (green), LamB (white), and Cnn (red). White arrowheads indicate the position of centrosomes. Scale bars: 5 μm. (F) Bar graph quantification of the percentage of ovarioles per ovary attached to at least two contiguous egg chambers (ECs)*.* The number of ovaries analyzed is indicated at the top of the bars. **P<0.01, ****P<0.0001. (G) Box plots of the size of centrosomal Cnn in interphase GSCs of the indicated genotype. For each box plot, the box represents the 25th-75th percentile interval, the line represents the median, and the whiskers represent the 5th-95th percentile interval and non-outlier range. The number of centrosomes analyzed is indicated above each box. Statistical analysis used the unpaired, two-sample *t*-test. *P<0.1, ****P<0.0001. (H) Bar graph quantification of the percentage of GSCs with centrosomes (marked by Cnn) showing α-Tubulin enrichment. The number of GSCs analyzed is indicated at the top of the bars. Statistical analysis used the unpaired, two-sample *t*-test. ****P<0.0001. ns, not significant.

The phenotypic rescue of *emerin* mutants with reduced PCM levels suggests that abnormal centrosomes contribute to loss of GSC homeostasis. Therefore, we tested whether an unscheduled activation of interphase centrosomes was sufficient to block germ cell differentiation and promote GSC loss. To this end, we used an approach similar to one used previously to prevent centrosome disassembly in oocytes (Pimenta-Marques et al., 2016). In our studies, we overexpressed GFP-tagged variants of Polo (Fig. 4A), the mitotic kinase that drives centrosome maturation (Conduit, 2024). The *nanos* (*nos*) *gal4* driver provided germline-restricted overexpression of either an inactive kinase [Polo-kinase dead (KD)-GFP] or a constitutively active (CA) kinase (Polo-CA-GFP; Fig. 4A). Western blot analysis of protein extracts obtained from *nos>polo-KD-GFP* and *nos>polo-CA-GFP* ovaries established that responder transgenes produced a single prominent band of the expected molecular weight (Fig. 4B, Fig. S3), with Polo-KD-GFP accumulated at approximately 4-fold higher levels than Polo-CA-GFP (Fig. 4C).

We next set out to determine the effects of ectopic expression of Polo-GFP derivatives. To this end, newly eclosed ovaries were stained with DAPI and antibodies against GFP. Notably, the majority of *nos>polo-KD-GFP* (99%) and *nos>polo-CA-GFP* (91%) ovarioles were associated with strands of differentiating GFP-positive egg chambers (Fig. 4D-F), indicating that expression of either Polo variant permits robust oogenesis. Further, both *nos>polo-KD-GFP* and *nos>polo-CA-GFP* females laid eggs that hatched. We wondered whether the absence of a phenotypic effect, especially in the *nos>polo-CA-GFP* ovaries, was because these conditions failed to generate abnormal interphase centrosomes. To test this prediction, *nos>polo-KD-GFP* and *nos>polo-CA-GFP* ovaries were stained with antibodies against Cnn and α-Tubulin. Strikingly, the size of Cnn foci in interphase *nos>polo-CA-GFP* GSCs was nearly double that of either *nos>polo-KD-GFP* or wild-type GSCs (0.99 µm versus 0.44 µm and 0.49 µm, respectively; Fig. 4D,E,G), mirroring the approximate twofold increase in size of the Cnn foci in *emerin* mutants relative to wild-type controls (Duan et al., 2021). Whereas few *nos>polo-KD-GFP* or wild-type Cnn foci were enriched with microtubules (15% and 13%, respectively; Fig. 4D,E,H), microtubules were found at nearly all (97%) interphase *nos>polo-CA-GFP* Cnn foci, a degree of enrichment higher than found at *emerin* mutant Cnn foci (∼35%; Fig. 3B). These findings demonstrate that Polo-CA-GFP promotes formation of centrosomes carrying excess PCM in interphase GSCs. Surprisingly, these centrosomes did not affect nuclear structure, evidenced by LamB staining (Fig. 4E). These data indicate that nucleation of microtubules at the nuclear periphery does not alter nuclear structure, aligning with our findings that elimination of PCM recruitment to centrioles in *cnn, emerin* or *emerin; spd-2* mutant germ cells did not suppress nuclear distortion (Fig. 3C). We conclude that ectopic production of activated interphase centrosomes alone fails to cause germ cell dysfunction.

### Combined NL and centrosome dysfunction causes loss of GSC homeostasis

**Fig. 5.**
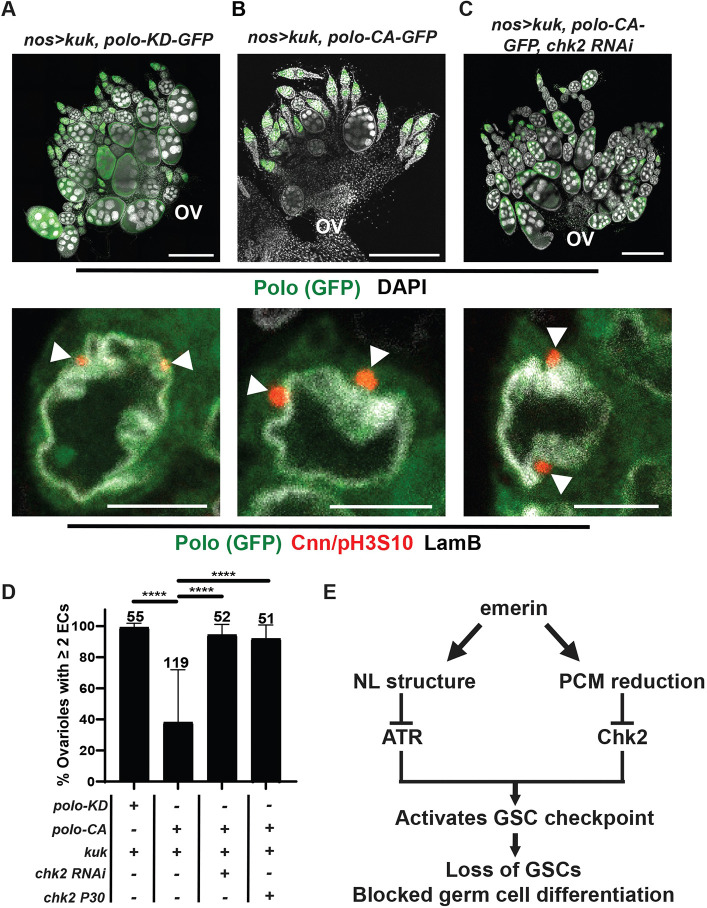
**Combined centrosome dysfunction and NL distortion causes loss of GSC homeostasis.** (A-C) Top: Confocal images of NE ovaries of the indicated genotype stained with antibodies against GFP (Polo; green) and DAPI. Ovaries are oriented so that anterior (germaria) is at the top and posterior (oviduct, ov) is at the bottom. Note that GFP staining is restricted to germ cells. Bottom: Confocal images of NE GSC nuclei stained with antibodies against GFP (Polo, green), Cnn (red), pH3S10 (red), and LamB (white). Interphase GSCs are pH3S10 negative. White arrowheads indicate the position of centrosomes. Scale bars: 5 μm. (D) Bar graph quantification of the percentage of ovarioles per ovary attached to at least two contiguous egg chambers (ECs)*.* Genotypes are indicated in the column below each bar. ‘+’ indicates the presence and ‘−’ indicates the absence of the indicated transgene or mutant allele. The number of ovaries analyzed is indicated at the top the bars. Statistical analysis used the unpaired, two-sample *t*-test. ****P<0.0001. (E) Model integrating effects of NL dysfunction and defects in PCM reduction. We propose that Emerin promotes both NL structural integrity and PCM reduction. In the absence of Emerin, NL distortion occurs, leading to ATR activation, and centrosome enlargement occurs, leading to increased accumulation of Chk2 at interphase centrosomes. Enhancing the proximity of interphase centrosomal Chk2 to activated ATR triggers the GSC checkpoint. As a result, GSCs are lost and germ cell differentiation is blocked.

In our studies, we noted a correlation between NL deformation and the loss of GSC homeostasis. In *cnn, emerin* or *emerin; spd-2* mutant GSCs, NL distortion remained and germ cell function was not completely restored (Fig. 3C), whereas in *nos>polo-CA-GFP* GSCs there was no NL distortion and germ cell function was unperturbed (Fig. 4E). For this reason, we wondered whether loss of GSC homeostasis in *emerin* mutants resulted from combined centrosome and NL defects. This was tested by simultaneous production of enlarged centrosomes and NL distortion in genetically wild-type GSCs. NL distortion was produced using the *UASp-kuk* responder transgene, which overexpresses the permanently farnesylated NL protein Kugelkern (Kuk), a rate-limiting factor for nuclear growth (Brandt et al., 2006; Petrovsky et al., 2018). Overexpression of Kuk induces severe nuclear distortion in GSCs without effects on oogenesis (Fig. S4; Perales et al., 2024a). First, we generated recombinant lines carrying either *UASp-kuk* and *UASp-Polo-CA-GFP* or *UASp-kuk* and *UASp-Polo-KD-GFP* responder transgenes. Next, animals from the recombinant responder lines were crossed with the animals from the *nos-gal4* driver line. Newly eclosed ovaries from resulting progeny were stained with DAPI and antibodies against GFP to detect germ cell-restricted Polo expression. Strikingly, dual expression of Kuk and Polo-CA-GFP, but not dual expression of Kuk and Polo-KD-GFP, disrupted germ cell development in most ovaries (Fig. 5A,B). Quantification established that the vast majority (99%) of ovarioles in *nos>kuk, polo-KD-GFP* ovaries had strings of differentiating egg chambers, whereas fewer (38%) ovarioles had strings of differentiating egg chambers in *nos>kuk, polo-CA-GFP* ovaries (Fig. 5D). Next, we examined interphase nuclear phenotypes of *nos>kuk, polo-KD-GFP* and *nos>kuk, polo-CA-GFP* GSCs by staining with antibodies against GFP, Cnn, pH3S10 and LamB. Strikingly, nuclear distortion occurred in both genotypes, but only *nos>kuk, polo-CA-GFP* GSC nuclei had enlarged Cnn foci (Fig. 5A,B). Notably, these enlarged Cnn foci localized to positions of greatest nuclear ruffling (Fig. 5A,B). Based on these findings, we conclude that production of interphase centrosomes with excess PCM in wild-type GSCs when combined with severe nuclear distortion promotes GSC loss and blocks germ cell differentiation.

To understand whether the GSC NL pathway was activated in *nos>kuk, polo-CA-GFP* ovaries, we examined effects of Chk2 reduction. To this end, we generated *nos>kuk, polo-CA-GFP, chk2RNAi* animals that carried a *chk2 RNAi* responder transgene, as well as *chk2^+/−^, nos>kuk, polo-CA-GFP* animals that were heterozygous for a null allele of *chk2*. In both genetic backgrounds, oogenesis was restored, evident by findings that 94% of *nos>-kuk, polo-CA-GFP, chk2RNAi* and 92% of *chk2^+/−^, nos>kuk, polo-CA-GFP* ovarioles carried at least two contiguous differentiating egg chambers, respectively (Fig. 5C,D). Restoration of oogenesis did not result from reduction of excess PCM on centrosomes or reversal of nuclear distortion because both defects remained in *nos>kuk, polo-CA-GFP, chk2RNAi* ovaries (Fig. 5C). These observations indicate that the combination of nuclear distortion and abnormal interphase centrosomes are upstream of, and contribute to, Chk2 activation.

## DISCUSSION

### Centrosome dysfunction and NL distortion activate the GSC checkpoint

NL proteins safeguard stem cell maintenance. Indeed, loss of Emerin activates a GSC checkpoint that leads to premature stem cell loss. Here, we show that elimination of active interphase centrosomes rescued GSC survival and promoted early stages of differentiation in *emerin* mutants (Figs 1, 2), implicating involvement of dysfunctional centrosomes in loss of GSC homeostasis. Even so, elimination of enlarged centrosomes did not completely suppress Chk2 activation (Fig. S2) and ectopically produced abnormal interphase centrosomes did not disrupt GSC homeostasis (Fig. 4). Together, these findings demonstrate that abnormal centrosomes contribute to, but are not the only input required for, activation of the GSC NL checkpoint in *emerin* mutants.

NL deformation was a common feature of unhealthy GSCs (Fig. 3), even though structural changes in the NL alone failed to promote stem cell loss (Fig. S2). These observations suggested that NL distortion might represent a second input for checkpoint activation. For this reason, we induced abnormal centrosomes and NL distortion in genetically wild-type GSCs. Remarkably, these GSCs were prematurely lost and germ cell differentiation was strongly reduced (Fig. 5), conditions that reconstituted phenotypes associated with Emerin loss. Although the effects on germ cell development were severe, the presence of some differentiating oocytes differs from phenotypes seen in *emerin* mutants (Fig. 5D), suggesting that Emerin might have additional roles in germ cell development. Indeed, a small class of genes has been identified that appear to require Emerin for transcription in the ovary (Kitzman et al., 2022). Notably, the defects caused by induction of abnormal centrosomes and NL distortion are suppressed by genetic reduction of Chk2 (Fig. 5). We conclude that checkpoint activation in *emerin* mutants requires two inputs (Fig. 5E): incomplete PCM removal to form abnormal centrosomes and nuclear distortion.

A recent study of a human infantile dilated cardiomyopathy found that impaired centrosome reduction caused cardiomyocyte-specific defects due to a block in reorganization of the microtubule network needed for development (Chun et al., 2023). These findings predict that abnormal centrosomes in *emerin* mutants have the potential to disrupt the microtubule network needed for GSC self-renewal and germ cell differentiation. However, we found that oogenesis is robust in the presence of enlarged microtubule-enriched centrosomes in wild-type GSCs (Fig. 4E,F), suggesting that GSCs tolerate at least some level of reorganization of the microtubule network.

### Links between checkpoint activation and the DDR kinases

Our studies reveal that two inputs are required for activation of the GSC checkpoint. We propose that these inputs are linked to the two DDR kinases involved in this checkpoint pathway (Fig. 5E): ATR and Chk2 (Barton et al., 2018). Although an ATR-to-Chk2 signaling axis differs from the canonical ATM (Tefu)-to-Chk2 or ATR (Mei-41)-to-Chk1 (Grp) axes (Burgess and Misteli, 2015), persistent meiotic double-strand breaks activate ATR and Chk2 in fly and mouse germlines (Abdu et al., 2002; Bolcun-Filas et al., 2014; Klattenhoff et al., 2007), suggesting that ATR-to-Chk2 signaling might be commonly used in germ cells.

Multiple triggers can activate ATR (Burgess and Misteli, 2015; Kidiyoor et al., 2016; Marechal and Zou, 2013). In addition to DNA damage, ATR senses topological and mechanical forces at the nuclear envelope (Bermejo et al., 2011; Kumar et al., 2014). As ATR activation in *emerin* mutant GSCs lacks signatures of the canonical DDR, NL deformation has been suggested to be a likely trigger (Barton et al., 2018). Indeed, mechanical activation of ATR depends upon its recruitment to the NL by the Rad9-Hus1-Rad1 (9-1-1) complex (Kumar et al., 2014; Lanz et al., 2018). In *Drosophila*, the major determinant of the 9-1-1 complex localization is Rad9, a protein that resides in the nuclear membrane of germ cells (Kadir et al., 2012). Based on these observations, we predict that nuclear distortion in GSCs promotes Rad9, and thereby 9-1-1 clustering, facilitating ATR recruitment and activation (Fig. 5E). Even so, without a second input, we predict that this ATR activation is ineffective at blocking germ cell function. The second kinase is Chk2. Notably, Chk2 localizes to centrosomes during mitosis (Takada et al., 2003), a localization that is independent of DNA damage. Centrosome targeting of Chk2 is facilitated by its forkhead-associated domain that binds phospho-epitopes (Takada et al., 2015). In our studies, expression of catalytically active, but not inactive, Polo kinase activated Chk2 (Fig. 5E). We suggest that large amounts of phospho-epitopes in the expanded PCM elevates levels of Chk2 at the centrosome. We envision that these high levels of Chk2 increase its physical proximity to activated NL ATR, which promotes Chk2 phosphorylation and its activation (Fig. 5E). Alternatively, it remains possible that ectopic centrosomes might recruit sufficient levels of Chk2 for ATR-independent activation, as Chk2 overexpression alone can promote its activation (Bakhrat et al., 2010). However, we do not favor this model because Chk2 overexpression in GSCs has no effect on their survival (Bakhrat et al., 2010), a finding that aligns with our data that ectopic production of centrosomes alone does not promote stem cell loss (Fig. 4). Taken together, we suggest that NL-activated ATR and centrosome-enriched Chk2 collaborate to trigger a GSC checkpoint.

### Connections between Emerin and centrosomes are conserved

Emerin contributes to the reduction of PCM in interphase centrosomes in GSCs (Fig. 3B). Two observations suggest that these effects are direct. First, *Drosophila* Emerin is a bona fide component of centrosomes (Habermann et al., 2012; Muller et al., 2010), where it interacts with γ-Tubulin and α-Tubulin (Habermann et al., 2012). Second, *Drosophila* Emerin has roles in centrosome maturation and cell cycle progression during embryogenesis, with evidence that loss of Emerin contributes to the regulation of centrosome size (Habermann et al., 2012). Notably, human emerin also interacts with β-tubulin-containing centrosomes and controls centrosome positioning within cells (Salpingidou et al., 2007). Further, a recent proteome-wide comparison of the interactome for three human LEM-domain proteins revealed that the emerin-specific interactome is enriched for centrosomal- or microtubule-associated proteins (Moser et al., 2020). These observations, coupled with our findings, suggest that Emerin might have a conserved function in centrosome regulation. Deregulation of centrosome structure is implicated in many human diseases (Arquint et al., 2014). We suggest that diseases resulting from mutations in genes encoding NL components might represent another class of disorders linked to centrosome dysfunction.

## MATERIALS AND METHODS

### *Drosophila* stocks

*Drosophila* stocks were raised on standard cornmeal/agar medium with p-hydroxybenzoic acid methyl ester as a mold inhibitor. In all analyses, the reference strain is *y^1^w^67c23^* (wild type; Bloomington Stock Center, #6599). The *emerin* mutant refers to *y^1^w^67c23^; ote^B279G/PK^*, in which the *ote^B279G^* allele carries an insertion of a Piggybac transposon at +764 (Bloomington Stock Center, #16189) and the *ote^PK^* carries a premature stop at codon 127 (Barton et al., 2013). The *cnn* mutant background refers to *cnn^HK21/mfs3^*, in which the *cnn^HK21^* allele carries a premature stop at codon 106 and *cnn^mfs3^* carries a premature stop at codon 959 (Li et al., 1998; Megraw et al., 1999). To generate *cnn, emerin* double mutants, recombinants were identified by sequencing PCR fragments generated using primers shown in Table S1. The *spd-2* mutant background refers to *spd-2^Z-5711/Df(3L)^*^BSC561^, in which *spd-2^Z-5711^* carries a premature stop at codon 56 (Giansanti et al., 2008) and *spd-2^DF(3L)BSC561^* carries a deletion that includes the *spd-2* gene (Bloomington Stock Center, #25123). The *chk2* mutant background refers to *chk2^+/p30^*, in which the *chk2^p30^* allele carries a deletion of the 5′-UTR and first two coding exons (LaRocque et al., 2007). All alleles used in these studies are null alleles. Crosses carrying germline alleles were conducted at 22°C. In our *GAL4-UAS* studies, the germline driver used in these studies was *P[GAL4:VP16-nos]* (Bloomington Stock Center, #4937). The Polo responder lines were *pUASp-PoloT182D-GFP*, which expresses a GFP tagged full-length Polo variant that has constitutive kinase activity (CA), and *pUASp-PoloK54M-GFP*, which expresses a GFP-tagged, full-length Polo variant that lacks kinase activity (KD); responder transgenes were integrated into the *attP154* site (3R, 97D2; Kachaner et al., 2017), generously provided by Vincent Archambault (University of Montreal, Canada). Crosses between driver and Polo responder lines were conducted at 25°C. The Kuk responder line was *UASp-kuk*, which expresses full-length Kugelkern, inserted at the *attP2* site (3L, 68A4; Perales et al., 2024a). Crosses between driver and this responder line were conducted at 22°C. Additional information about the genes studied can be found at FlyBase (Grumbling and Strelets, 2006; Jenkins et al., 2022; Ozturk-Colak et al., 2024).

### Immunohistochemical analysis

Ovaries from newly eclosed (<1 day old) or aged (14 days old) females were dissected in cold PBS solution, as described by Duan et al. (2023). Briefly, dissected ovaries were fixed at room temperature in 4% EM grade paraformaldehyde (Electron Microscopy Sciences, 15710), washed in PBST (PBS with 0.3% Triton X-100) three times for 5 min each wash and blocked in 5% w/v bovine serum albumin in PBST at room temperature for 1 h. For studies involving analysis of microtubules, ovaries were dissected and fixed in room temperature PBS. Primary antibodies were added for incubation overnight at 4°C. Subsequently, tissues were washed in PBST three times for 10 min each wash and incubated with Alexa Fluor-conjugated secondary antibodies (at 1:500) at room temperature for 2 h. Secondary antibodies were: donkey anti-goat 488 (A11055), donkey anti-mouse 488 (A21202), donkey anti-mouse 568 (A10037), donkey anti-mouse 647 (A31571), donkey anti-rabbit 568 (A10042) and donkey anti-rabbit 647 (A31573) (all from Molecular Probes). After additional washes in PBST, tissues were mounted in SlowFade (Thermo Fisher Scientific). Confocal images were collected with a Leica SP8 confocal microscope and processed using ImageJ imaging software. Primary antibodies were: mouse anti-Engrailed (1:50; Developmental Studies Hybridoma Bank, 4D9); mouse anti-Spectrin (1:50; Developmental Studies Hybridoma Bank, 3A9); rabbit anti-Vasa (1:500; provided by Zissimos Mourelatos, University of Pennsylvania, PA, USA); mouse anti-α-Tubulin (1:1000; Millipore, DM1A); mouse anti-Lamin B (1:300; DS Developmental Studies Hybridoma Bank, HB ADL84.12); rabbit anti-Plp (1:1000; provided by Greg Rogers, University of Arizona, AZ, USA); rabbit anti-Centrosomin (1:10,000; provided by Nasser Rusan, NHLBI, NIH, MD, USA); rabbit anti-phosphoserine10 Histone 3 (1:1000; Abcam, 47297); goat anti-GFP (1:2000; Abcam, 6673); rabbit anti-Asterless (1:1000; provided by Nassar Rusan). For each immunohistochemical-based analysis, germaria were imaged from at least five ovaries obtained from at least two biological replicates. In every experiment, control wild-type and *emerin* mutant ovaries were stained and imaged alongside experimental ovaries.

### Quantification and statistical analysis

Several methods were used for quantification of ovary phenotypes. (1) The percentage of germ-cell-containing germaria per ovary was quantified from ovaries stained with antibodies against Engrailed and Vasa. Germ cell-containing germaria were defined as Vasa-positive germaria. Statistical analysis was performed in GraphPad Prism using the unpaired, two-sample *t*-test. (2) The amount of germ cell differentiation was quantified from ovaries stained with antibodies against Spectrin and Vasa. Differentiation was scored as germaria that carried germ cells with a round spectrosome and germ cells with branched fusomes. Using these criteria, the percentage of germaria per ovary showing branched fusomes was determined. Statistical analysis was performed in GraphPad Prism using the unpaired, two-sample *t*-test. (3) The percentage of differentiated ovarioles was quantified by counting the number of ovarioles per ovary that contained at least two contiguous egg chambers beyond the germarium. Statistical analysis was performed in GraphPad Prism using the unpaired, two-sample *t*-test. (4) The sizes of the interphase PCM was determined from ovaries stained with Cnn antibodies. *z*-sections of confocal images of Cnn staining were scanned to identify the mid-section of the signal. Foci were traced and the size defined using ImageJ (v.1.54f; National Institutes of Health). Statistical analysis was performed in GraphPad Prism using the unpaired, two-sample *t*-test. (5) Nucleation of microtubules was determined from ovaries stained with α-Tubulin and a centriole/PCM protein antibody. *z*-sections of confocal images were scanned to identify the mid-section of the signal. We scored a positive enrichment if the α-Tubulin signal in the section overlapped with, and emanated from, the centriole/PCM signal. Statistical analysis was performed in GraphPad Prism using the unpaired, two-sample *t*-test.

### Western blot analysis

Polo levels were assessed using proteins extracted from ovaries dissected from newly eclosed females. Proteins were electrophoresed on a 4-12% gradient Tris gel and blotted onto a nitrocellulose membrane. Membranes were probed with primary antibodies against rabbit anti-GFP (1:1000; Abcam, 290) or rat anti-Vasa (1:1000, Developmental Studies Hybridoma Bank, AB 760351). Primary antibodies were detected using Alexa Fluor-conjugated secondary antibodies [donkey anti-rat 680, 1:10,000 (LI-COR, 926-68073); donkey anti-rabbit 800, 1:10,000 (Invitrogen, A32808)].

## Supplementary Material

10.1242/develop.204219_sup1 Supplementary information
