# Supplementary material for: Emerin preserves stem cell survival through maintenance of centrosome and nuclear lamina structure
Source: Development. 2024 Nov 13;151(22):dev204219. doi: 10.1242/dev.204219 (PMC11586520; doi:10.1242/dev.204219)
Supplement: Supplementary information [file develop-151-204219-s1.pdf]

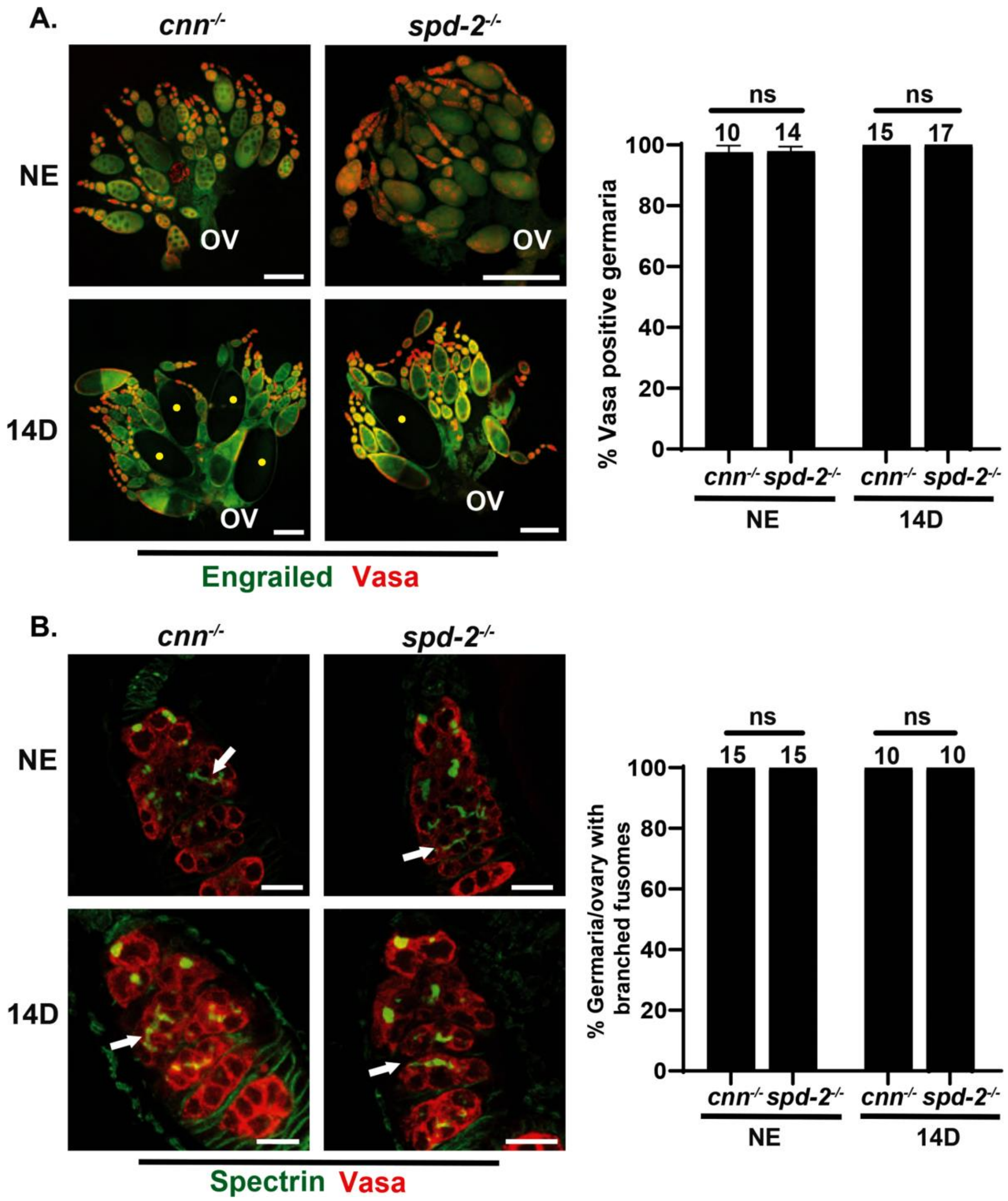

Fig. S1. Cnn and Spd-2 are dispensable for oogeneis.

A. Left: Confocal images of *cnn*$^{-/-}$ or spd-2$^{-/-}$ NE (top) and 14-day-old (bottom) ovaries stained with antibodies against Engrailed (green) and Vasa (red). Yellow circles show late-stage egg chambers. OV indicates the ovaduct of the ovary. Scale bars, 200µm. Right: Bar graph quantification of

Development • Supplementary information

the percentage of germ cell-containing niches. The number of ovaries analyzed is indicated at the top of the bars. Statistical analysis used the unpaired two sample t-test. ns= not significant. **B.** Left: Confocal images of *cnn*$^{-/-}$ or spd-2$^{-/-}$ NE (top) and 14-day-old (bottom) ovaries stained with antibodies against Spectrin (green) and Vasa (red). White arrows show spectrin in what is considered differentiated. Scale bars 1Oμm. Right: Bar graph quantification of the percentage of germaria exhibiting germ cell differentiation (branched Spectrin localization). The number of ovaries analyzed is indicated at the top of the bars. Statistical analysis used the unpaired two sample t-test. ns= not significant.

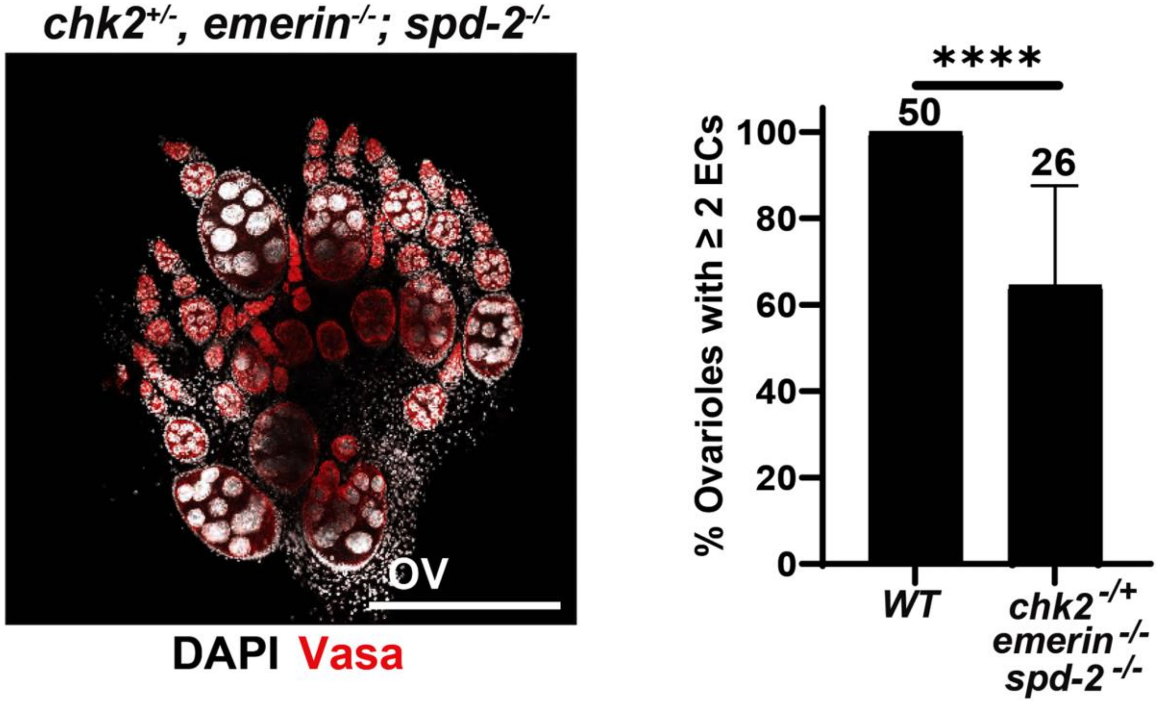

Fig. S2. Chk2 remains active upon PCM reduction in emerin mutants.

Left: Confocal image of a *chk2*$^{+/-}$, *emerin*$^{-/-}$*; spd-2*$^{-/-}$ ovary stained with antibodies against DAPI (white) and Vasa (red). OV indicates the ovaduct of the ovary. Scale bars, 200µm. Right: Bar graph quantification of the percentage of ovarioles with ≥2 egg chambers (ECs). The number of ovaries analyzed is indicated at the top of the bars. Statistical analysis used the unpaired two sample t-test. Asterisks indicate significance ****<.0001.

Development • Supplementary information

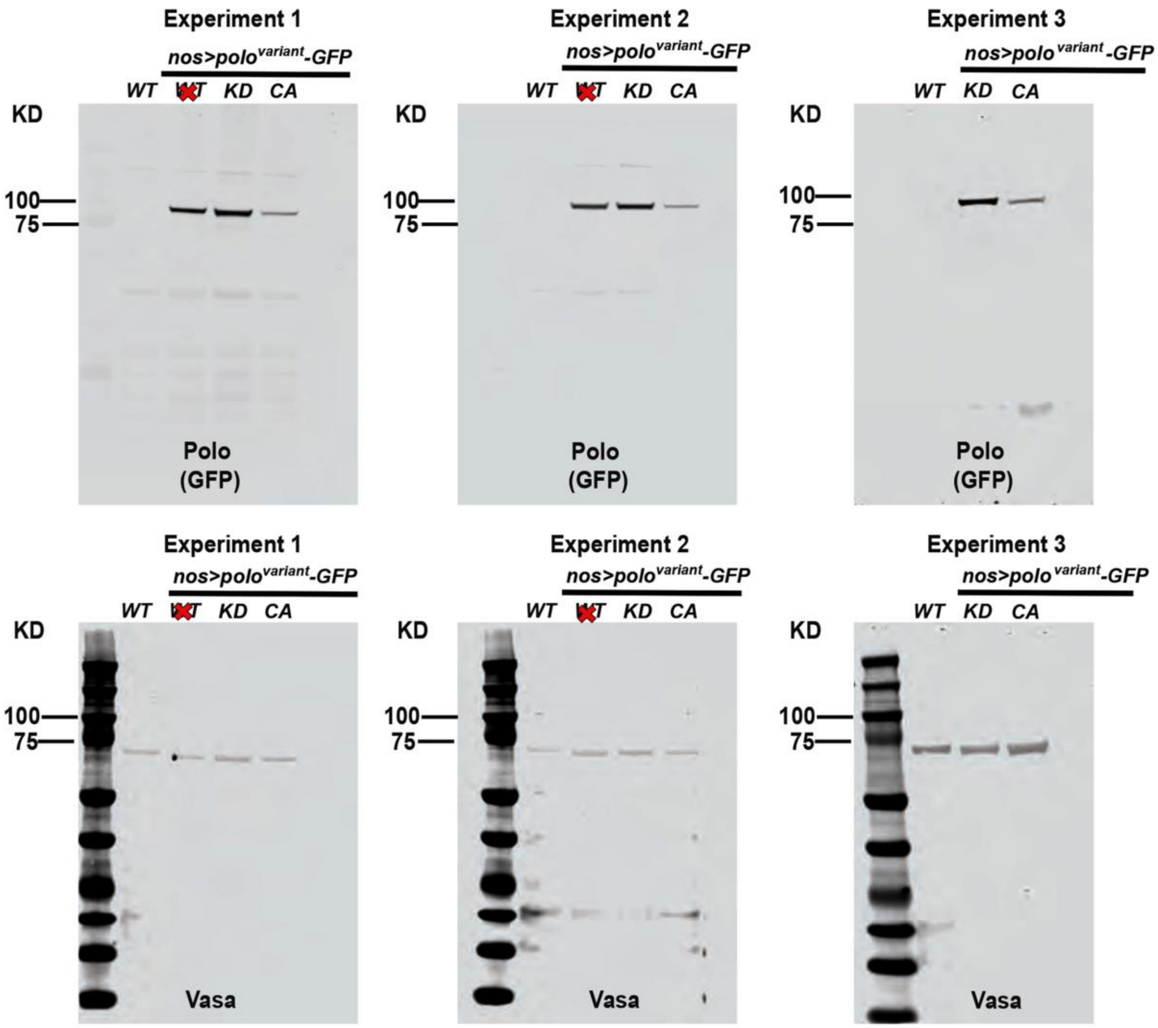

	Fold Expression to Polo-KD			
	Exp. 1	Exp. 2	Exp. 3	Average
Polo-KD	1	1	1	1
Polo-CA	0.189	0.226	0.263	0.226

Fig. S3. Western blot analysis of expression of Polo variants in the ovary. Shown are three western blots from three experiments that extracted proteins of newly eclosed ovaries obtained from female progeny of distinct parents. Blots were probed with antibodies against GFP (Polo, top) and Vasa (bottom) . The size of the 100- and 75-kD molecular weight marker is shown at the left. Below western blots is a table summarizing the fold expression of *Polo-CA-GFP* relative to *the Polo-KD-GFP* variant.

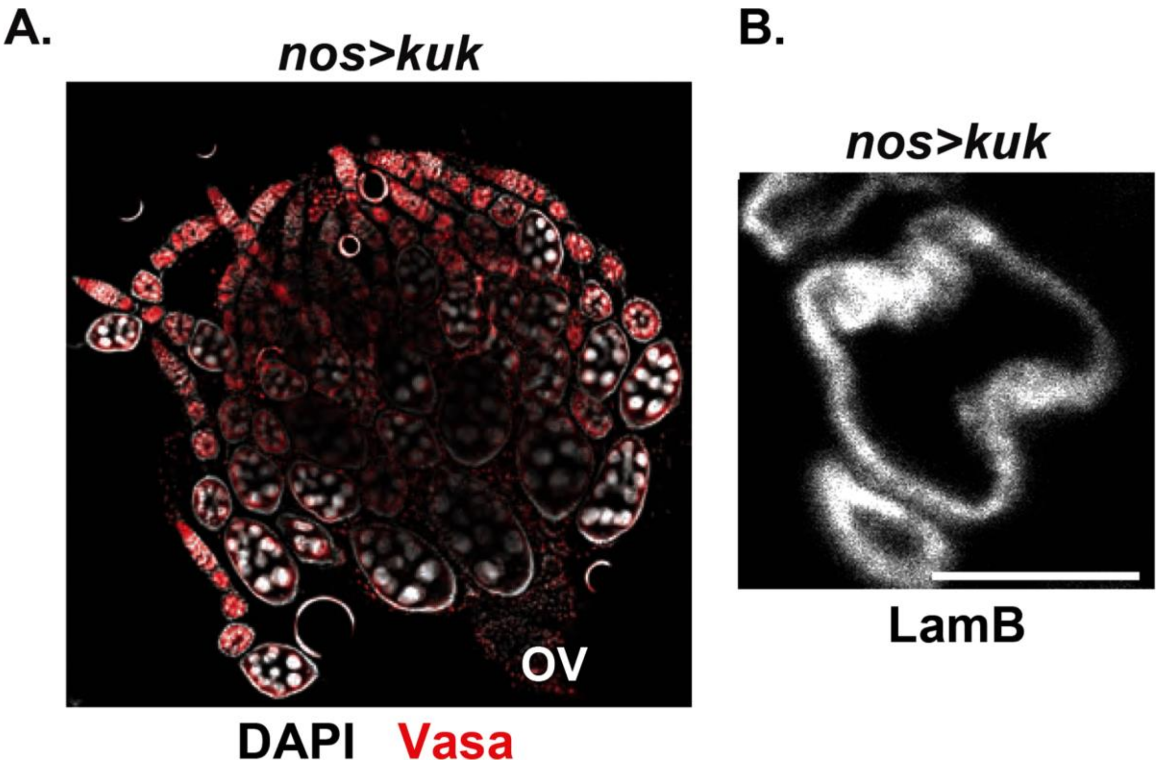

Fig. S4. Oogenesis is robust in the presence of GSC nuclear distortion caused by Kuk overexpression.

A. Shown is a confocal image of a *nos>kuk* NE ovary stained with antibodies against DAPI (white) and Vasa (red). OV indicates the ovaduct of the ovary**. B.** Confocal image of a GSC nuclei stained with antibodies against LamB (white), showing severe NL distortion. Scale bars, 5µm.

Table S1. DNA primers used for genotyping

**Primers (5' to 3')**		
**Allele**	**Forward primer**	**Reverse primer**
cnn^{HK21}	TTGACGTTCCACGACCTCCAG	TGAATTCCAGCTCGTTCCGGG
cnn^{mfs3}	ACCCTGCAATCCCAGCTACAG	GCAGCCTGTTTGCACGTTGTA
ote^{pk}	GGATCCATGGCCGATGTGGAC	GGATCCTCAGTAGAATATGTAATAAACGCCGATTAAC

Development • Supplementary information
